# Interesting Case of Suspension of Growth of a Fœtus in Utero, at about the Seventh Month of Gestation—Its Subsequent Death and Delivery at Full Term

**Published:** 1867-10

**Authors:** S. D. Mercer

**Affiliations:** Omaha, Neb.


ARTICLE XLVI.

### INTERESTING CASE OF SUSPENSION OF GROWTH OF A FŒTUS IN UTERO, AT ABOUT THE SEVENTH MONTH OF GESTATION—ITS SUBSEQUENT DEATH AND DELIVERY AT FULL TERM.

BY S. D. MERCER, M.D., of Omaha, Neb.

Mrs. A., *æt.* 32; healthy; in ordinary circumstances. Removed from Michigan, by car, to this city, in January, about the third month of pregnancy. She has had three previous normal labors, and three abortions, two of which were induced.

*May 23.* She worked very hard washing, although seven months and ten days advanced. The following morning she woke up, weeping, and told her husband that her child was dead, in the womb. Previous to this, she had been perfectly well, and she continued to do some house work up to the very day of her confinement. Though naturally of a cheerful disposition, she became melancholy and uneasy.

*June 10.* I was called to the case. The abdomen was soft and tympanitic, and she said it was much smaller than it had been for several weeks previous to the change. The fundus of the uterus could be felt above the umbilicus. There was no tenderness; no constipation of bowels, nor any discharge from the vagina. The mouth of the womb could not be reached by an ordinary digital examination. The breast was secreting copiously. She had felt no movements of the child since May 23d, except "a slight rising of the womb." By careful auscultation I detected slight, but unmistakable, fœtal pulsation. I told them I regarded it as a case of suspension of the growth of the fœtus, depending upon the shock received by the hard day's work; that the child was certainly living then, but probably would not be much longer.

*June 11.* A slight discharge of water, tinged with blood, occurred, not very much, but enough to exclude all possibility of mistake.

*July 7.* A similar discharge occurred, and continued, in a small quantity, for two days.

*July 10.* Dr. Morrell saw the case, with me. She has felt no movements, whatever, for ten days. It is now about the completion of full term. After a careful examination, Dr. M. confirmed the previous diagnosis, but no fœtal pulsation could then be detected.

*July 13.* She fell in labor at half-past 9 in the evening, and about 2 the following morning was delivered of a dead child.

The child was evidently one of about seven months growth. Sutures were not united, and this rendered the labor very easy. The pains came on very frequent, but were light. The waters were discharged copiously a few minutes before the labor was completed. There was considerable decomposition of the cuticle of the child, but not enough to cause any unnatural odor.

The placenta came away very easily. It was small, and partially decomposed on the surface that was attached to the womb. There was considerable fatty degeneration of the cord. The mother had a very good getting up, with no untoward symptoms, except an unusual discharge of the lochia, and a subsequent purulent discharge, which has not entirely ceased yet.

